# Heat shock protein 90 mediates the protective effects of vericiguat on myocardial ischemia/reperfusion injury by inhibiting toll-like receptor 4 and c-Jun N-terminal kinases

**DOI:** 10.22038/ijbms.2025.84354.18248

**Published:** 2025

**Authors:** Si-Jie Pan, Jun-Yan Chen, Dong-Xiao Wang, Jian-Jun Meng, Min Wang, Guo-Qiang Zhong, Zhi-Yu Zeng, Rong-Hui Tu

**Affiliations:** 1 Department of Cardiology, First Affiliated Hospital of Guangxi Medical University, Nanning 530021, Guangxi, China; 2 Department of Geriatric Cardiology, First Affiliated Hospital of Guangxi Medical University, Nanning 530021, Guangxi, China; 3 Guang Xi Key Laboratory of Precision Medicine in Cardio-cerebrovascular Disease Control and Prevention, Nanning 530021, Guangxi, China; 4 Guangxi Clinical Research Center for Cardio-Cerebrovascular Diseases, First Affiliated Hospital of Guangxi Medical University, Nanning 530021, Guangxi, China

**Keywords:** HSP90, Ischemic postconditioning Ischemic preconditioning JNK, TLR4, Vericiguat

## Abstract

**Objective(s):**

This study aimed to investigate whether vericiguat exerts a protective effect against myocardial ischemia-reperfusion injury (MIRI) by inhibiting toll-like receptor 4 (TLR4) and c-Jun N-terminal kinases (JNK) activation and whether heat shock protein 90 (HSP90) mediates these effects.

**Materials and Methods:**

A total of 120 male mice were randomly divided into six groups: sham, ischemia/reperfusion (I/R group), VPreC (vericiguat, 3 mg/kg, administered intravenously 12 hr before ligation), VPreC+HSP90 inhibitor geldanamycin (GA) (geldanamycin, 1 mg/kg, injected intraperitoneally 30 min before ligation), VPostC (vericiguat, 3 mg/kg, administered intravenously ten minutes before reperfusion), and VPostC+GA (geldanamycin, 1 mg/kg, injected intraperitoneally 20 min before reperfusion). The remaining five groups were subjected to 30 min of ischemia followed by two hours of reperfusion. The sizes of myocardial infarction, rates of cardiomyocyte apoptosis, and levels of myocardial markers were measured. In addition, the protein expressions of HSP90, TLR4, JNK, BAX, and B-lymphoblastoma-2 (Bcl-2) were detected, along with the mRNA levels of inflammatory factors.

**Results:**

Vericiguat significantly reduced I/R-induced myocardial infarct size, apoptosis rate, and myocardial marker release. Alongside these positive effects, there was an increase in HSP90 and Bcl-2 expression, as well as a decrease in TLR4, JNK, BAX expression, and inflammatory factor levels. However, the HSP90 inhibitor GA reversed these protective and anti-inflammatory effects.

**Conclusion:**

HSP90 mediates the cardioprotective effects of vericiguat, potentially by inhibiting TLR4, JNK activation, and inflammatory responses.

## Introduction

Cardiovascular disease remains the leading cause of death worldwide, with acute myocardial infarction (AMI) constituting the primary driver of cardiovascular-related mortality (1, 2). Despite significant advances in the diagnosis and treatment of acute coronary syndromes, achieving effective reperfusion of the myocardium remains a central strategy for therapy. However, the rapid reperfusion process is often accompanied by an inescapable dilemma: myocardial ischemia reperfusion injury (MIRI), a process that may further exacerbate myocardial damage (3). Therefore, exploring and developing safe and efficient therapeutic approaches to attenuate or prevent such reperfusion injury is crucial for improving patients’ prognoses with MIRI.

The soluble guanylate cyclase (sGC) agonist vericiguat has gained increasing interest from scholars in the field of cardiovascular therapy (4, 5). Vericiguat’s efficacy has been shown to extend beyond heart failure with reduced ejection fraction (HFrEF) and may exhibit positive signs of attenuating MIRI. Recent studies indicate that vericiguat intervention reduces myocardial infarct size, prevents apoptosis, and enhances cardiac function after MIRI (6, 7). Despite the promising results of these studies, further thorough and methodical research is necessary to understand the precise molecular mechanisms.

Heat shock protein 90 (HSP90) is an abundant and highly sequence-conserved molecular chaperone protein that maintains proteins’ correct folding and conformational maturation, thereby preserving intracellular protein homeostasis (8). Several experimental studies have demonstrated HSP90’s essential role in mitigating MIRI, and it is a key effector molecule in the significant anti-MIRI effects of both ischemic preconditioning (IPC) and ischemic postconditioning (IPostC), which are effective endogenous protective mechanisms in ischemic myocardium (9-12). Wang *et al*. found that HSP90 participates in the resistance of anesthetic preconditioning to MIRI (13). Additionally, Chen *et al*. proposed that IPC can protect against lung ischemia reperfusion injury in rabbits by increasing the expression of HSP90 (14). HSP90’s effects on mitigating MIRI have also been well-established in previous studies (10, 15-17). However, whether HSP90 plays a role in the protective effects of vericiguat in MIRI remains unclear. 

Toll-like receptor 4 (TLR4), which is widely present on cardiomyocyte membranes, is essential for inducing inflammatory and apoptotic cascade responses in MIRI. It can promote the expression of inflammatory factors, initiating and mediating local inflammatory reactions, which leads to cardiac injury (18, 19). A study has shown that the activation of TLR4, along with its related signal transduction pathway and inflammatory response, plays a crucial role in MIRI, and that TLR4 suppression dramatically decreases markers of MIRI and inflammatory response (20). Notably, HSP90 may contribute to IPostC-mediated cardiac protection by blocking TLR4 activation (15). Nevertheless, whether the interaction between HSP90 and TLR4 is involved in vericiguat-induced cardioprotection is unknown.

As members of the mitogen-activated protein kinases (MAPK) family, c-Jun N-terminal kinases (JNKs) play a role in regulating inflammation and the signaling pathways of apoptosis and necrosis (21). They are activated in response to various stress stimuli, including heat shock, oxidative stress, and ischemia-reperfusion injury in the brain and heart (22). Research has shown that HSP90 attenuates ischemia reperfusion (I/R)-induced myocardial injury and apoptosis through the inhibition of JNK (16, 17). Hu *et al*. found that the cardioprotective effects of α-toco in a mouse MIRI model involved the joint participation of HSP90, Bcl-2, and JNK signaling pathways (23). Moreover, research has demonstrated that exogenous biglycan protects myocardial cells from ischemia-reperfusion injury via TLR4-mediated mechanisms involving the activation of JNK (24). 

Numerous studies have highlighted the significance of HSP90, TLR4, and JNK in mitigating MIRI (15-17, 23, 24). However, it remains unclear whether the cardioprotective effects of vericiguat are associated with HSP90, TLR4, or JNK, and whether HSP90 mediates the cardioprotective effect of vericiguat by inhibiting TLR4, blocking JNK activation, and reducing inflammatory responses. Therefore, in this study, we established a mouse ischemia-reperfusion model to assess the cardioprotective effects of vericiguat by measuring the myocardial infarction area, the apoptosis rate of cardiomyocytes, and the levels of myocardial markers such as creatine kinase isoenzyme (CK-MB), lactate dehydrogenase (LDH), and cardiac troponin I (cTnI). Western blot analysis was utilized to measure the protein expression levels of HSP90, TLR4, JNK, BAX, and B-lymphoblastoma-2 (Bcl-2). RT-qPCR was used to quantify the mRNA levels of interleukin (IL)-6, intercellular adhesion molecule-1 (ICAM-1), and tumor necrosis factor-α (TNF-α) to examine the inflammatory response. We also introduced the HSP90 inhibitor geldanamycin (GA) to investigate its impact on the intervention effect of vericiguat and to explore the role of HSP90 in the protective mechanism of vericiguat. 

## Materials and Methods

### Animals

Pathogen-free grade C57/BL6 mice (male, 8-10 weeks old, body weight 22±2 g) were provided by the Laboratory Animal Center. The animals were housed in a standard 12-hour light–dark cycle environment at 25±2 ^°^C and 50±15% humidity, having free access to food and water. The experimental protocols used in this study adhered to the National Research Council’s Guide for the Care and Use of Laboratory Animals (25). They were authorized by the Animal Protection and Use Committee. The animals’ welfare was maintained throughout the study by ensuring humane treatment and integrating their well-being into the planning and execution of every process during the research period (26). 

### Myocardial I/R models

The mice were anesthetized with an intraperitoneal injection of sodium pentobarbital (50 mg/kg). Their tracheas were then intubated and ventilated with positive pressure (100 breaths/min) using a small animal ventilator (KW-100-2, KEW BASIS, Nanjing, China). The electrical activity levels of the mice’s hearts were monitored with an electrocardiograph (DE06, DAWEI, Jiangsu, China) linked to their limbs. Their chests were subsequently opened at the left third intercostal space to fully expose the heart. The left anterior descending (LAD) branch of the coronary artery was crossed with an 8-0 suture line 1 mm below the inferior border of the left atrial appendage. The end of the suture line was passed through a small plastic tube to create a reversible LAD occlusion. After 30 min of tightening the ligature around the plastic tube, myocardial ischemia was induced by compressing the LAD. The ligature was then released, reversing the occlusion for two hours, allowing for reperfusion. An electrocardiogram (ECG) ST-segment elevation and myocardial whitening in the LAD blood supply indicated that the model was successfully constructed (27). Following reperfusion, the mice were euthanized, and cardiac tissue and blood samples were extracted from the anterior wall of the left ventricle near the apical region for further analysis.

### Experimental groups

**Figure 1:**
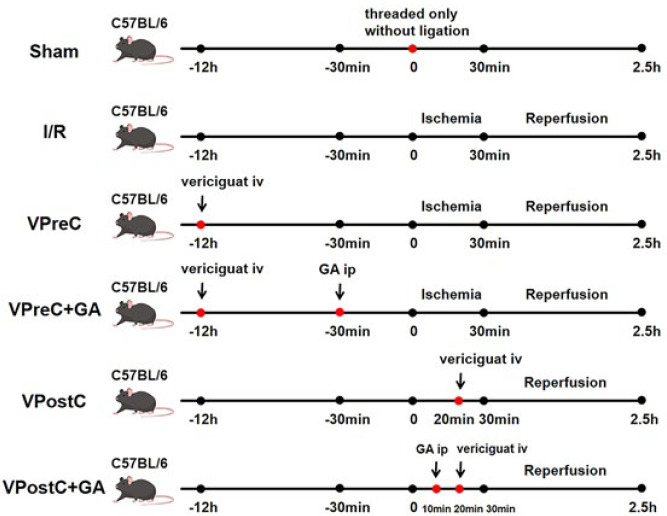
Schematic diagrams of different experimental treatments for each group

The sample size of 120 mice was determined by referring to the pre-experiment results and associated data analysis, along with a review of the literature (28-31). These mice were randomly divided into the following six groups (N=20) using basic randomization software (Microsoft Co., Redmond, USA): (1) sham group-LAD was threaded only without ligation for 150 min; (2) I/R group-underwent ligation of the LAD for 30 min and then reperfusion for 2 hr; (3) VPreC group-underwent ligation of the LAD for 30 min, followed by reperfusion for 2 hr, and intravenous administration of vericiguat (3 mg/kg (32)) for 12 hr before the ligation; (4) VPreC+GA group-intraperitoneal injection of GA (1 mg/kg (10)) was given 30 min before ligation, while the rest of the operation remained the same as that of the VPreC group; (5) VPostC group-ligation of the LAD for 30 min, followed by reperfusion for 2 hr, and intravenous administration of vericiguat (3 mg/kg (32)) 10 min before the reperfusion; (6) VPostC+GA group-intraperitoneal injection of GA (1 mg/kg (10)) was administered 20 min before reperfusion, with the rest of the operation remaining the same as that of the VPostC group (Figure 1).

### Determination of myocardial infarct size

After reperfusion, the LAD was ligated again, and a 1% Evans blue staining solution (DK0050, Leagene Biotechnology, Beijing, China) was injected into the inferior vena cava (33). The stained hearts were sectioned and sliced to a thickness of 0.8 mm after being frozen (34). Subsequently, processing with 1% 2,3,5-triphenyl tetrazolium chloride (TTC) (DK0004, Leagene Biotechnology, Beijing, China) for 15 min at 37 ^°^C was performed, and the myocardial infarct area and total left ventricular area were quantified using Image J software (v1.8.0.345, National Institutes of Health, Bethesda, USA).

### Levels of CK-MB, LDH, and cTnI in serum

Following reperfusion, blood samples were collected, and serum was extracted by centrifuging (5424R, Eppendorf, Hamburg, Germany) the samples for 15 min at 3000 rpm. CK-MB and LDH levels were determined using a fully automated biochemical analyzer (Catalyst One, IDEXX Laboratories Inc., Maine, USA). cTnI levels were measured using a cTnI ELISA kit (KT80246, MSK, Wuhan, China) on an autoanalyzer (7600, Hitachi, Tokyo, Japan).

### Terminal deoxynucleotidyl transferase-mediated dUTP-X nick end labeling (TUNEL) staining

After dewaxing and hydrating the paraffin sections of cardiac tissues, the sections were stained using a terminal deoxynucleotidyl transferase-mediated dUTP-X nick end labeling (TUNEL) detection kit (C1091, Beyotime, Shanghai, China)(35). Cell staining was observed under a microscope (400x magnification, BX53, OLYMPUS, Tokyo, Japan). The nuclei of apoptotic cells (TUNEL-positive cells) were stained brown, while the nuclei of normal cardiomyocytes were stained blue. At least five fields of view containing apoptotic cells were randomly selected. The cells were quantitatively analyzed using Image J software (v1.8.0.345, National Institutes of Health, Bethesda, USA), and the apoptotic index was expressed as the ratio of TUNEL-positive cells to total cardiomyocytes multiplied by 100%.

### RNA extraction and RT qPCR

Total RNA was extracted from myocardial tissues of the ischemic area using an RNA-easy Isolation Reagent (R701, Vazyme Biotech Co., Ltd., Nanjing, China). The extracted RNA was then quantified with a nano-microdroplet spectrophotometer (840-317500, Thermo Fisher Scientific, Massachusetts, USA), and the obtained RNA was reverse transcribed using a HyperScript Reverse Transcription Kit (R202, EnzyArtisan, Shanghai, China). Polymerase chain reactions were performed with the SYBR ® Green qPCR Mix Kit (Q204, EnzyArtisan, Shanghai, China) on an ABI Prism 7500 System (NO.275051604, Thermo Fisher Scientific, Massachusetts, USA). Forty-five cycles of thermal cycling were conducted at 95 ^°^C for 30 sec, 95 ^°^C for 10 s, and 60 ^°^C for 30 sec. The housekeeping gene used was 3-Phosphoglyceraldehyde dehydrogenase (GAPDH). Relative mRNA expression was analyzed using the 2-ΔΔCT method (36). The primers used were as follows:

GAPDH gene: 

forward: 5’-AGGTTGTCTCCTGCGACTTCA-3’, 

reverse: 5’-GGGTGGTCCAGGGTTTCTTA-3’.

IL-6 gene: 

forward: 5’-ACGGCCTTCCCTACTTCACA-3’, 

reverse: 5’-CTGCAAGTGCATCATCGTTGT-3’. 

ICAM-1 gene: 

forward: 5’-TGGGTCGAAGGTGGTTCTTCT-3’, 

reverse: 5’-GTCCAGCCGAGGACCATACAG-3’. 

TNF-α gene: 

forward: 5’-ACCACGCTCTTCTGTCTACTG-3’, 

reverse: 5’-GAGGGTCTGGGCCATAGAACT-3’.

### Western blot

Myocardial tissue samples were spiked with RIPA tissue lysate (PC101, Epizyme, Shanghai, China), processed using a tissue homogenizer (LUKYM-I/LU, LUKYM, Guangzhou, China), and then centrifuged at 4 ^°^C at 12,000 rpm for 15 min (5424R, Eppendorf, Hamburg, Germany). The supernatant was collected, and protein concentrations were determined using the BCA Protein Assay Kit (PC0020, Servicebio, Wuhan, China). Proteins in equal amounts were electrophoresed on SDS-PAGE gels (Mini-PROTEAN Tetra, Bio-Rad, California, USA) and subsequently transferred to nitrocellulose (PVDF) membranes (ISEQ00010, Merck, Darmstadt, Germany). These membranes were then blocked with 5% skim milk (P0216-300g, Beyotime, Shanghai, China) for one hour at room temperature and divided into strips based on the molecular weights of the target proteins, which were incubated with the corresponding primary antibodies: anti-GAPDH (Cat.No.GB150004, 1:4000 Servicebio, Wuhan, China), anti-HSP90 (Cat.No.13171-1-AP, 1:5000 Proteintech, Wuhan, China), anti-TLR4 (Cat.No.505258, 1:1000 Zenbio, Chengdu, China), anti-JNK (Cat.No.ab208035, 1:2000 Abcam, Cambridge, UK), anti-Bcl-2 (Cat.No.#40639, 1:2000 Signalway Antibody, Baltimore, USA), and anti-BAX (Cat.No.CY5059, 1:2000 ABWays, Shanghai, China). The incubation process was performed on a shaker overnight at 4 ^°^C. After three washes with TBST, the membranes were incubated in horseradish peroxidase-labeled goat anti-rabbit IgG antibody (Cat.No.S0001, 1:10000 Affinity, Jiangsu, China) for one hour at room temperature. Finally, the membranes were exposed to a chemiluminescent substrate (BL523B, Biosharp, Anhui, China), and the bands were detected using an infrared imaging system (NO.032217090131, e-BLOT, Shanghai, China). Protein expression was quantified using Image J software (v1.8.0.345, National Institutes of Health, Bethesda, USA)(37).

### Statistical analysis

IBM SPSS software (27.0, IBM Corp., Armonk, USA) was utilized to analyze the data, which were expressed as mean±standard deviation (X̄±s). A one-way variance analysis (ANOVA) was used to compare multiple groups’ means. *P*<0.05 was considered statistically significant.

## Results

The present study used 120 mice. Four mice were excluded due to cardiogenic shock during reperfusion and respiratory failure: two from the VPreC group, one from the I/R group, and one from the VPostC+GA group. The results presented here correspond to the 116 mice included in the study.

### Vericiguat up-regulated the expression of HSP90

**Figure 2:**
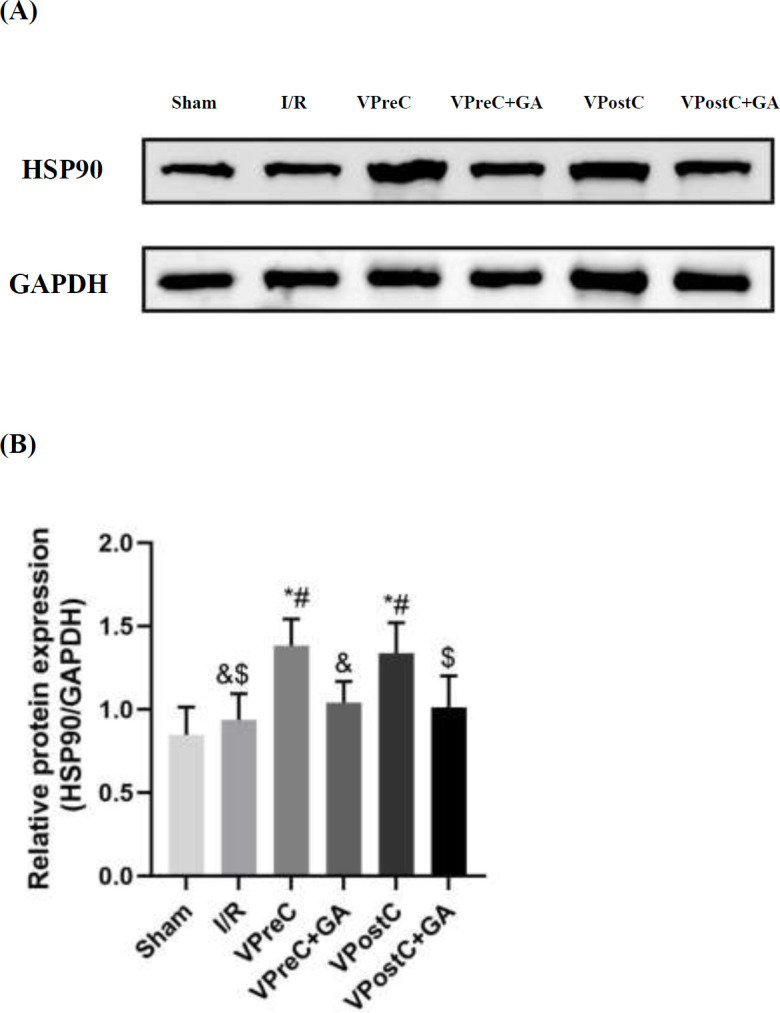
Effects of Vericiguat and Vericiguat+geldanamycin (GA) on HSP90 protein expression levels after myocardial ischemia/reperfusion injury in mice

As shown in Figure 2, HSP90 protein expression was higher in the VPreC group compared to the I/R and VPreC+GA groups (*P*<0.05, ANOVA, F=8.757). Additionally, HSP90 protein expression in the VPostC group was greater than in the I/R and VPostC+GA groups (*P*<0.05, ANOVA, F=8.757). The VPreC+GA and VPostC+GA groups did not show a statistically significant difference from the I/R group (*P*>0.05, ANOVA, F=8.757). These results suggest that vericiguat up-regulates HSP90 expression and that GA treatment counteracts the effects of vericiguat.

### Vericiguat reduces I/R-induced myocardial infarction area via HSP90

**Figure 3:**
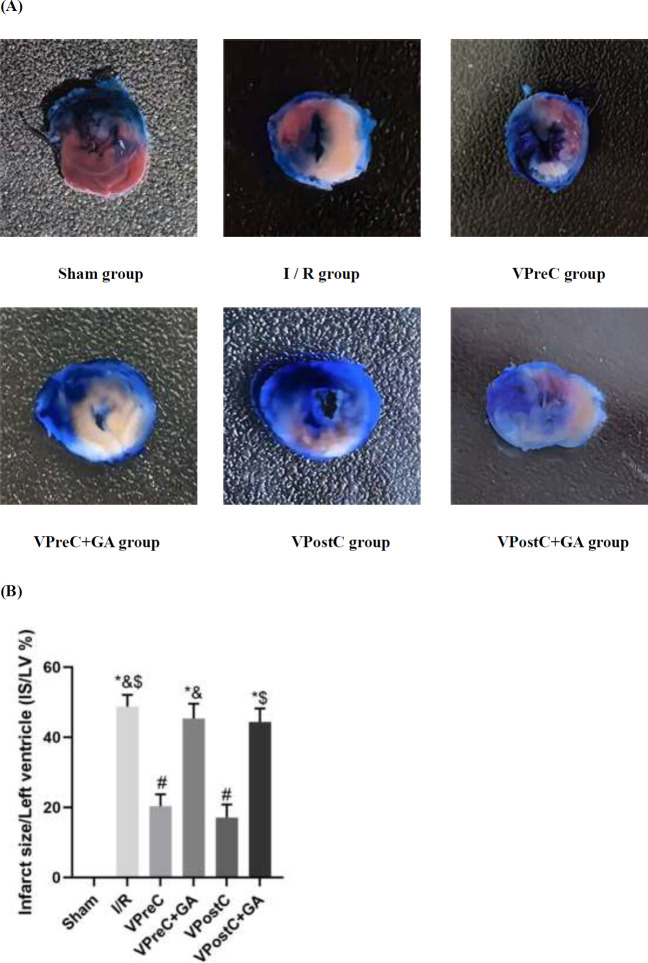
Effect of Vericiguat and Vericiguat+geldanamycin (GA) on myocardial infarct area after myocardial ischemia-reperfusion injury in mice

As shown in Figure 3, myocardial infarction did not occur in the sham group. Infarction area was reduced in the VPreC group compared with the I/R and the VPreC+GA groups (*P*<0.05, ANOVA, F***=***168.433) and reduced in the VPostC group compared with the I/R and the VPostC+GA groups (*P*<0.05, ANOVA, F***=***168.433). The VPreC+GA and VPostC+GA groups were not statistically different from the I/R group (*P*>0.05, ANOVA, F***=***168.433). The results suggest that vericiguat reduced I/R-induced myocardial infarction area via HSP90, whereas GA reversed the protective effect of vericiguat.

### Vericiguat attenuates I/R-induced cardiomyocyte apoptosis via HSP90

**Figure 4:**
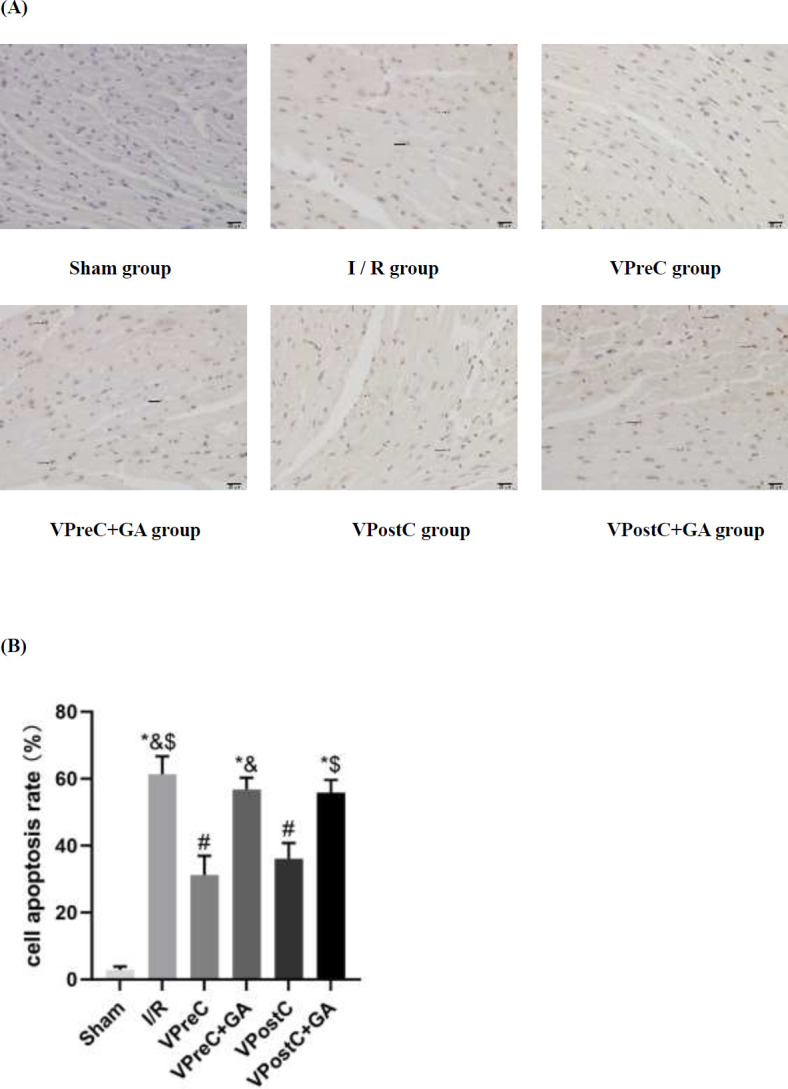
Effect of Vericiguat and Vericiguat+geldanamycin (GA) on apoptosis after myocardial ischemia-reperfusion injury in mice (×400 magnification, bar=20 μm)

Figure 4 shows that the level of cardiomyocyte apoptosis in the I/R group was significantly higher than that in the sham group (*Ρ<*0.05, ANOVA, F***=***133.778). The rate of cardiomyocyte apoptosis in the VPreC group was significantly lower than in the I/R and VPreC+GA groups (*Ρ<*0.05, ANOVA, F***=***133.778). In addition, the apoptosis rate of cardiomyocytes in the VPostC group was significantly lower than that in the I/R and the VPostC+GA groups (*Ρ<*0.05, ANOVA, F***=***133.778). The VPreC+GA and VPostC+GA groups were not statistically different from the I/R group (*Ρ>*0.05, ANOVA, F***=***133.778). The results suggest that Vericiguat attenuated I/R-induced cardiomyocyte apoptosis via HSP90, while GA reversed Vericiguat’s apoptosis-inhibitory effect.

### Vericiguat attenuates I/R-induced myocardial injury via HSP90

Table 1 indicates that the CK-MB (1470.0±184.4 vs 372.4±40.0; *P*<0.05, ANOVA, F***=***65.359), LDH (2201.5±248.0 vs 755.8±82.0; *P*<0.05, ANOVA, F***=***67.484), and cTnI (42.3±5.6 vs 4.8±1.0; *P*<0.05, ANOVA, F***=***68.707) levels of the I/R group were significantly higher than those of the sham group. CK-MB (536.8±59.4 vs 1470.0±184.4 and 1461.6±251.4; *P*<0.05, ANOVA, F***=***65.359), LDH (1206.6±153.7 vs 2201.5±248.0 and 2209.6±176.7; *P*<0.05, ANOVA, F***=***67.484), and cTnI (15.9±2.6 vs 42.3±5.6 and 44.8±7.4; *P*<0.05, ANOVA, F***=***68.707) levels were significantly lower in the VPreC group compared to the I/R and VPreC+GA groups. CK-MB (682.0±90.0 vs 1470.0±184.4 and 1665.4±232.2; *P*<0.05, ANOVA, F***=***65.359), LDH (1344.8±192.9 vs 2201.5±248.0 and 2298.6±169.2; *P*<0.05, ANOVA, F***=***67.484), and cTnI (14.1±3.1 vs 42.3±5.6 and 36.4±4.5; *P*<0.05, ANOVA, F***=***68.707) levels were significantly lower in the VPostC group compared with the I/R and the VPostC+GA groups. The VPreC+GA and VPostC+GA groups were not statistically different from the I/R group (*Ρ*>0.05, ANOVA, F***=***65.359; 67.484; 68.707). The results indicate that vericiguat attenuated I/R-induced myocardial injury via HSP90, whereas GA reversed the inhibitory effect of vericiguat on I/R-induced myocardial injury.

### Vericiguat attenuates I/R-induced inflammatory response via HSP90

**Figure 5:**
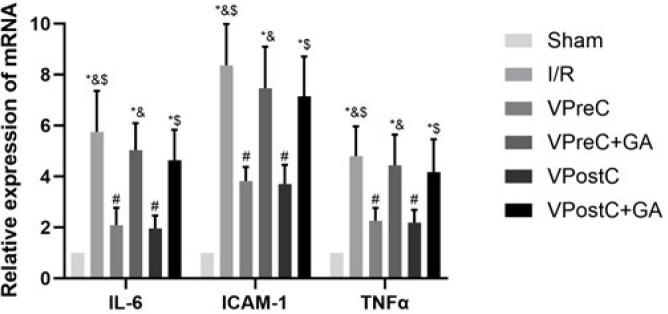
Effects of Vericiguat and Vericiguat+geldanamycin (GA) on mRNA levels of IL-6, ICAM-1, and TNF-α after myocardial ischemia/reperfusion injury in mice

As shown in Figure 5, compared with the sham group, the IL-6, ICAM-1, and TNF-α levels were elevated in the I/R group (*P*<0.05, ANOVA, F***=***31.135; 72.392; 14.136). The IL-6, ICAM-1, and TNF-α levels were lower in the VPreC group compared to the I/R and VPreC+GA groups (*P*<0.05, ANOVA, F***=***31.135; 72.392; 14.136). The IL-6, ICAM-1, and TNF-α levels were lower in the VPostC group compared to the I/R and VPostC+GA groups (*P*<0.05, ANOVA, F***=***31.135; 72.392; 14.136). The VPreC+GA and VPostC+GA groups were not statistically different from the I/R group (*P*>0.05, ANOVA, F***=***31.135; 72.392; 14.136). The results suggest that vericiguat attenuated the I/R-induced inflammatory response via HSP90 and that GA treatment reversed the anti-inflammatory effect of vericiguat.

### Vericiguat attenuates the expression of TLR4 and JNK via HSP90

**Figure 6:**
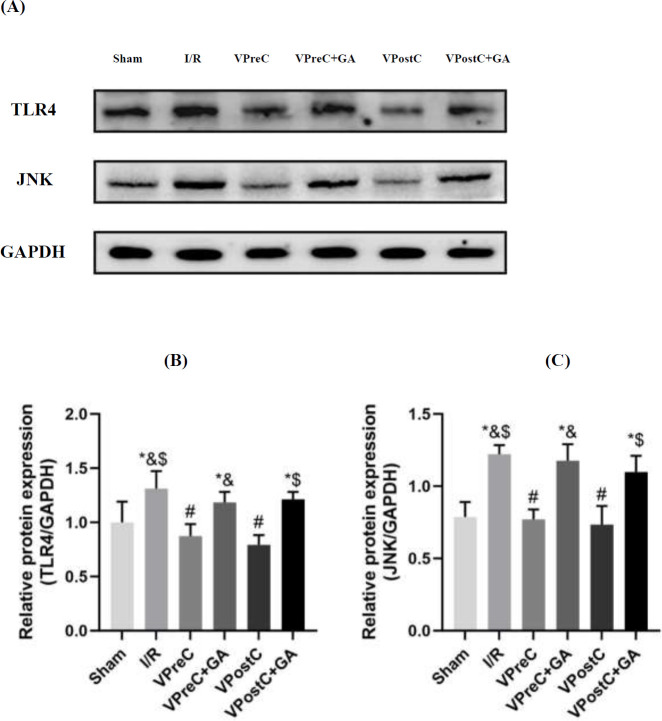
Effects of Vericiguat and Vericiguat+geldanamycin (GA) on TLR4 and JNK protein expression levels after myocardial ischemia/reperfusion injury in mice

As shown in Figure 6, the TLR4 and JNK expression levels were higher in the I/R group than in the sham group (*P*<0.05, ANOVA, F***=***13.046; 24.431). The TLR4 and JNK protein levels in the VPreC group were lower than those in the I/R and VPreC+GA groups (*P*<0.05, ANOVA, F***=***13.046; 24.431). The levels of TLR4 and JNK proteins in the VPostC group were lower than those in the I/R and VPostC+GA groups (*P*<0.05, ANOVA, F***=***13.046; 24.431). The VPreC+GA and VPostC+GA groups were not statistically different from the I/R group (*P*>0.05, ANOVA, F***=***13.046; 24.431). These results indicate that vericiguat decreased TLR4 and JNK expression via HSP90 and that GA treatment counteracted the effect of vericiguat.

### Effects of vericiguat and GA on BAX and Bcl-2 protein expression

**Figure 7:**
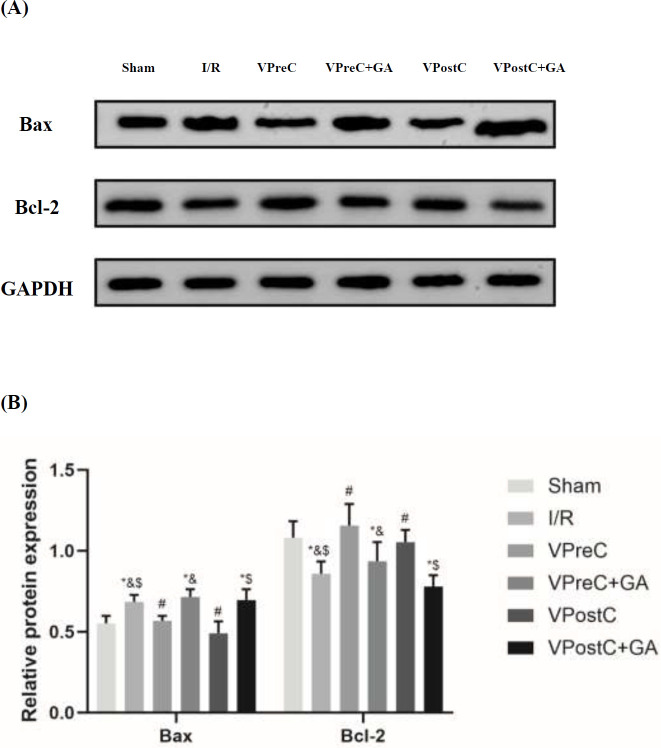
Effects of Vericiguat and Vericiguat+geldanamycin (GA) on Bax and Bcl-2 protein expression levels after myocardial ischemia/reperfusion injury in mice

As shown in Figure 7, the expression level of BAX was significantly elevated in the I/R group compared with the sham group (*P*<0.05, ANOVA, F***=***14.786). The BAX protein level in the VPreC group was lower than in the I/R and VPreC+GA groups (*P*<0.05, ANOVA, F***=***14.786). Similarly, the BAX protein level in the VPostC group was lower than the levels in the I/R and the VPostC+GA groups (*P*<0.05, ANOVA, F***=***14.786). The VPreC+GA and VPostC+GA groups were not statistically different from the I/R group in BAX expression. (*P*>0.05, ANOVA, F***=***14.786). The expression level of Bcl-2 was significantly reduced in the I/R group compared to the sham group (*P <*0.05, ANOVA, F***=***10.569). Moreover, the Bcl-2 protein level in the VPreC group was higher than that in the I/R and VPreC+GA groups (*P*<0.05, ANOVA, F***=***10.569). Lastly, the Bcl-2 protein level in the VPostC group was higher than those in the I/R and the VPostC+GA groups (*P*<0.05, ANOVA, F***=***10.569). The VPreC+GA and VPostC+GA groups were not statistically different from the I/R group in Bcl-2 expression. (*P*>0.05, ANOVA, F***=***10.569). The results indicate that vericiguat enhanced vericiguat’s anti-apoptotic effect via HSP90, whereas GA treatment reversed the same effect. 

## Discussion

The significant finding of this study was that vericiguat significantly decreased the I/R-induced activation of TLR4, JNK, and inflammatory responses through HSP90. The results show that vericiguat preconditioning and postconditioning reduced the area of I/R-induced myocardial infarction, rate of apoptosis, and release of myocardial markers (CK-MB, LDH, and cTnI); down-regulated the expression of TLR4, JNK, and BAX; up-regulated HSP90 and Bcl-2 expression; and simultaneously diminished the levels of inflammatory factors (IL-6, ICAM-1, TNF-α). The HSP90 inhibitor GA, however, counteracted these protective effects. These data indicate that vericiguat-induced cardioprotective benefits are HSP90-dependent.

Vericiguat is a novel sGC stimulator that numerous cardiovascular studies and clinical trials have confirmed to lower cardiovascular mortality, risk of heart failure hospitalization, and incidence of worsening events in heart failure (4, 5). The American College of Cardiology (ACC) and the European Society of Cardiology (ESC) both recommend vericiguat in their guidelines for patients with HFrEF who are at high risk of reduced ejection fraction after standardized diagnosis and treatment (38, 39). Recent studies have identified the potential value of vericiguat in attenuating MIRI. Cai *et al*. established a mouse model of MIRI and discovered that the myocardial infarction area in mice was reduced and that the mice’s myocardial microcirculation and cardiac function all greatly improved after treatment with vericiguat (6). Zhu *et al*. found that vericiguat attenuates MIRI in pigs by encouraging autophagy, inhibiting apoptosis, and stimulating angiogenesis (7). In the present study, we focused on investigating the possible effects of vericiguat on MIRI. Our data showed that vericiguat attenuated MIRI by reducing myocardial infarct size, mitigating cardiomyocyte apoptosis, lowering the release of myocardial markers (CK-MB, LDH, and cTnI) and inflammatory cytokines (IL-6, ICAM-1, TNF-α), promoting the protein expression of Bcl-2, and down-regulating the protein levels of BAX.  Despite these findings, the specific molecular mechanisms underlying vericiguat have yet to be thoroughly investigated.

Previous research has revealed that HSP90 involves pioglitazone (28), liraglutide (29), and morphine (40) preconditioning or postconditioning mediated cardioprotective effects. To elucidate the mechanism underpinning vericiguat’s cardioprotective effect, we studied the expression of HSP90 in mice after they were treated with vericiguat. Interestingly, we found that the protein expression of HSP90 was markedly up-regulated in vericiguat preconditioning and postconditioning. We also discovered that the overexpression of HSP90 significantly ameliorated I/R-induced infarct size, cardiomyocyte apoptosis, the release of myocardial markers, and inflammatory cytokines. Still, the cardioprotective effect of vericiguat was significantly reversed by the HSP90 inhibitor GA, strongly indicating that HSP90 plays a critical role in the cardioprotective effect of vericiguat.

HSP90 is an integral component of cellular machinery that plays a crucial role in maintaining protein homeostasis (41). HSP90 aids in stabilizing proteins against stress, thereby preventing protein degradation caused by MIRI and protecting the heart (42). HSP90 has been proven to play a critical role in pharmacological pre- and postconditioning–mediated cardioprotective effects (28, 29, 40). Our results demonstrate that both vericiguat preconditioning and postconditioning could significantly up-regulate the expression of HSP90, thereby effectively attenuating myocardial injury induced by ischemia-reperfusion. However, the underlying mechanisms regarding the vericiguat-induced cardioprotection of HSP90 remain elucidated.   

TLRs, an evolutionarily conserved family of the innate immune system, are the host’s first line of defense against microbial pathogens by recognizing pathogen-associated molecular patterns (PAMPs)(43). TLR4, an influential member of the TLR family, is expressed most in cardiomyocytes compared to other TLR members and is implicated in a range of vital immune responses (44). TLR4 activates the expression of several pro-inflammatory cytokine genes that play key roles in myocardial inflammation, particularly myocarditis, atherosclerosis, myocardial infarction, ischemia-reperfusion injury, and heart failure (45, 46). In MIRI, TLR4 activation initiates intracellular signal transduction. It induces cells to produce and release a multitude of inflammatory factors, such as IL-6, IL-1β, and TNF-α, which mediate the cascade of inflammatory responses, causing the necrosis of cardiomyocytes, an increase in myocardial infarction area, and a decrease in cardiac function (47). 

Mitogen-activated protein kinase JNK is a common pathway associated with oxidative stress and apoptosis. It can be triggered by ischemia and reperfusion, which is crucial to developing ischemic injury (48, 49). Since the activation of these kinases is typically a response to tissue stress or injury, active MAPK is maintained at relatively low levels in most tissues under normal physiological homeostasis (50). In the pathogenesis of MIRI, JNK is activated to promote apoptosis and inflammatory responses, and the inhibition of the JNK signaling pathway lowers the levels of apoptosis and oxidative stress, thereby protecting against MIRI (22, 51). Notably, JNK and TLR4 were found to be interconnected in MIRI. TLR4 promotes JNK phosphorylation and activates the JNK pathway, triggering an inflammatory response and causing myocardial injury. The inhibition of TLR4 led to a reduction in JNK phosphorylation and significantly reduced myocardial markers and inflammatory response (20, 52-54). 

According to prior research, HSP90 was involved in postconditioning cardioprotection by inhibiting TLR4, JNK signaling, and inflammation (15, 16). To our knowledge, no study has addressed HSP90-mediated vericiguat cardioprotection by inhibiting TLR4, JNK signaling, and inflammation. It remains unclear whether vericiguat exerts a similar effect as postconditioning in inhibiting TLR4, JNK signaling, and inflammation and whether HSP90 mediates these effects. In the present study, treatment with vericiguat had higher HSP90 levels and lower TLR4, and JNK protein levels, as well as lower inflammatory cytokine levels (IL-6, ICAM-1, TNF-α), than those in I/R group. Nonetheless, GA reversed vericiguat-mediated TLR4, JNK signaling, and inflammation down-regulation. 

Cardiomyocyte apoptosis is a major mechanism of MIRI and leads to a progressive loss of cardiomyocytes, further enlarges infarct size, and causes cardiac insufficiency (10). The Bcl-2 gene family plays a central role in regulating this process, not only by controlling pro-apoptotic (BAX) and anti-apoptotic (Bcl-2) expression—the effects of which have been demonstrated in pharmacological preconditioning and postconditioning-but also by Bcl-2/BAX ratio (55-58). The expression of Bcl-2 and BAX regulated by HSP90 in postconditioning has been identified previously (10). Furthermore, a study has demonstrated that the HSP90 inhibitor blocks the interaction between HSP90 and Bcl-2, resulting in apoptosis (59). We assessed the effects of vericiguat combined with GA on the expression of Bcl-2 and BAX and found that GA reversed vericiguat-induced up-regulation of Bcl-2 and promoted the expression of BAX, suggesting that vericiguat’s anti-apoptotic effect is linked to HSP90.

This study is, to the best of our knowledge, the first to investigate the mechanism of vericiguat in attenuating MIRI by inhibiting the innate immune inflammatory response and cardiomyocyte apoptosis, providing new insights on vericiguat-mediated cardioprotection, offering a novel strategy and potential therapeutic option for treating MIRI, and contributing to the further expansion of the possible uses for vericiguat by opening up new avenues for its therapeutic indications. This study has some limitations. First, we only preliminarily explored the possible molecular mechanisms of vericiguat’s cardioprotective effects; additional studies are needed to elucidate the detailed molecular mechanisms. Second, we used only the HSP90-specific inhibitor GA in our study; the over- or under-expression of HSP90 with viral vectors can be explored in subsequent research. Third, our current work only shows the protein expression of TLR4 and JNK in vericiguat pre- and postconditioning. By applying TLR4 and JNK inhibitors, we may be able to further explore the intricate mechanisms of TLR4 and JNK as well as their downstream signal transduction mechanisms. Moreover, further study is needed to investigate whether vericiguat conducted cardioprotection through other heat shock proteins, such as HSP60 and HSP70.

Sham: Threaded only without ligation for 150 min; The remaining five groups were subjected to 30 min of ischemia followed by two hours of reperfusion

Vericiguat preconditioning (VPreC): Intravenous administration of vericiguat 12 hr before the ligation; Vericiguat preconditioning+geldanamycin(VPreC+GA): Intraperitoneal injection of GA 30 min before ligation; Vericiguat postconditioning (VPostC): Intravenous administration of vericiguat 10 min before the reperfusion; Vericiguat postconditioning+geldanamycin(VPostC+GA): Intraperitoneal injection of GA 20 min before reperfusion. iv: intravenous injection; ip: intraperitoneal

(A) Western blot detection of HSP90 expression; (B) Relative levels of HSP90 protein expression. Results are expressed as mean±standard deviation, *Ρ<0.05 compared with the sham group; ^#^*Ρ<*0.05 compared with the ischemia/reperfusion (I/R) group; ^&^*Ρ<*0.05 compared with the Vericiguat preconditioning (VPreC) group; ^$^*Ρ<*0.05 compared with the Vericiguat postconditioning (VPostC) group; N = 5

(A) Representative images of 2,3,5-triphenyltetrazolium chloride staining of hearts from different groups. (B) Quantitative analysis of myocardial infarct area. Results are expressed as mean±standard deviation, *Ρ<0.05 compared with the sham group; #*Ρ<*0.05 compared with the ischemia/reperfusion (I/R) group; ^&^*Ρ<*0.05 compared with the Vericiguat preconditioning (VPreC) group; ^$^*Ρ<*0.05 compared with the Vericiguat postconditioning (VPostC) group, N=5

**Table 1:** Serum CK-MB, LDH, and cTnI levels in different groups of mice

**G** **roup**	**CK-MB (ng/mL)**	**LDH (mU/mL)**	**cTnI(pg/mL)**
**Sham**	372.4±40.0	755.8±82.0	4.8±1.0
**I/R**	1470.0±184.4^*&$^	2201.5±248.0^*&$^	42.3±5.6^*&$^
**VPreC**	536.8±59.4^#^	1206.6±153.7^#^	15.9±2.6^#^
**VPreC+GA**	1461.6±251.4^*&^	2209.6±176.7^*&^	44.8±7.4^*&^
**VPostC**	682.0±90.0^#^	1344.8±192.9^#^	14.1±3.1^#^
**VPostC+GA**	1665.4±232.2^*$^	2298.6±169.2^*$^	36.4±4.5^*$^

Results are expressed as mean±standard deviation, *Ρ<0.05 compared with the sham group; #*Ρ<*0.05 compared with the ischemia/reperfusion (I/R) group; ^&^*Ρ<*0.05 compared with the Vericiguat preconditioning (VPreC) group; ^$^*Ρ<*0.05 compared with the Vericiguat postconditioning (VPostC) group, N=5

CK-MB: Creatine kinase isoenzyme; LDH: Lactate dehydrogenase; cTnI: Cardiac troponin I

(A) Representative images of terminal deoxynucleotidyl transferase-mediated dUTP-X nick end labeling (TUNEL) staining of hearts from different groups; (B) Quantitative analysis of cardiomyocyte apoptosis rate. Results are expressed as mean±standard deviation, *Ρ<0.05 compared with the sham group; #*Ρ<*0.05 compared with the ischemia/reperfusion (I/R) group; ^&^*Ρ<*0.05 compared with the Vericiguat preconditioning (VPreC) group; ^$^*Ρ<*0.05 compared with the Vericiguat postconditioning (VPostC) group, N=5

Results are expressed as mean±standard deviation, *Ρ<0.05 compared with the sham group; #*Ρ<*0.05 compared with the ischemia/reperfusion (I/R) group; ^&^*Ρ<*0.05 compared with the Vericiguat preconditioning (VPreC) group; ^$^*Ρ<*0.05 compared with the Vericiguat postconditioning (VPostC) group, N=5

(A) Western blot detection of TLR4, JNK expression; (B) Relative levels of TLR4 protein expression; (C) Relative levels of JNK protein expression. Results are expressed as mean±standard deviation, *Ρ<0.05 compared with the sham group; #*Ρ<*0.05 compared with the ischemia/reperfusion (I/R) group; ^&^*Ρ<*0.05 compared with the Vericiguat preconditioning (VPreC) group; ^$^*Ρ<*0.05 compared with the Vericiguat postconditioning (VPostC) group; N=5

(A) Western blot detection of Bax and Bcl-2 expression; (B) Relative Bax and Bcl-2 protein expression levels. Results are expressed as mean±standard deviation, *Ρ<0.05 compared with the sham group; #*Ρ<*0.05 compared with the ischemia/reperfusion (I/R) group; ^&^*Ρ<*0.05 compared with the Vericiguat preconditioning (VPreC) group; ^$^*Ρ<*0.05 compared with the Vericiguat postconditioning (VPostC) group; N=5

## Conclusion

HSP90 plays a critical role in vericiguat-mediated cardioprotection, possibly by inhibiting TLR4, JNK signaling, and inflammatory responses. This reduces I/R-induced myocardial infarction and cardiomyocyte apoptosis. 

## Data Availability

The data used to support this study’s findings are included within the article. The datasets generated and analyzed during the current study are available from the corresponding author upon reasonable request.

## Declaration

We have not used any AI tools or technologies to prepare this manuscript.

## References

[1] Bergmark BA, Mathenge N, Merlini PA, Lawrence-Wright MB, Giugliano RP (2022). Acute coronary syndromes. Lancet.

[2] Barrère-Lemaire S, Vincent A, Jorgensen C, Piot C, Nargeot J, Djouad F (2024). Mesenchymal stromal cells for improvement of cardiac function following acute myocardial infarction: A matter of timing. Physiol Rev.

[3] Welt FGP, Batchelor W, Spears JR, Penna C, Pagliaro P, Ibanez B (2024). Reperfusion injury in patients with acute myocardial infarction: JACC scientific statement. J Am Coll Cardiol.

[4] Pieske B, Butler J, Filippatos G, Lam C, Maggioni AP, Ponikowski P (2014). Rationale and design of the SOluble guanylate Cyclase stimulatoR in heArT failurE Studies (SOCRATES). Eur J Heart Fail.

[5] Armstrong PW, Pieske B, Anstrom KJ, Ezekowitz J, Hernandez AF, Butler J (2020). Vericiguat in patients with heart failure and reduced ejection fraction. N Engl J Med.

[6] Cai Y, Zhang B, Shalamu A, Gao T, Ge J (2022). Soluble guanylate cyclase (sGC) stimulator vericiguat alleviates myocardial ischemia-reperfusion injury by improving microcirculation. Ann Transl Med.

[7] Zhu W, Ben Y, Shen Y, Liu W (2023). Vericiguat protects against cardiac damage in a pig model of ischemia/reperfusion. PLoS One.

[8] Nair SP, Sharma RK (2020). Heat shock proteins and their expression in primary murine cardiac cell populations during ischemia and reperfusion. Mol Cell Biochem.

[9] Kupatt C, Dessy C, Hinkel R, Raake P, Daneau G, Bouzin C (2004). Heat shock protein 90 transfection reduces ischemia-reperfusion-induced myocardial dysfunction via reciprocal endothelial NO synthase serine 1177 phosphorylation and threonine 495 dephosphorylation. Arterioscler Thromb Vasc Biol.

[10] Zhong GQ, Tu RH, Zeng ZY, Li QJ, He Y, Li S (2014). Novel functional role of heat shock protein 90 in protein kinase C-mediated ischemic postconditioning. J Surg Res.

[11] Griffin TM, Valdez TV, Mestril R (2004). Radicicol activates heat shock protein expression and cardioprotection in neonatal rat cardiomyocytes. Am J Physiol Heart Circ Physiol.

[12] Amour J, Brzezinska AK, Weihrauch D, Billstrom AR, Zielonka J, Krolikowski JG (2009). Role of heat shock protein 90 and endothelial nitric oxide synthase during early anesthetic and ischemic preconditioning. Anesthesiology.

[13] Wang C, Qiao S, Hong L, Sun J, Che T, An J (2020). NOS cofactor tetrahydrobiopterin contributes to anesthetic preconditioning induced myocardial protection in the isolated ex vivo rat heart. Int J Mol Med.

[14] Chen SF, Wang H (2009). An experimental study of protective effects of ischemic preconditioning and oxymatrine on lung ischemia reperfusion injury. Sichuan Da Xue Xue Bao Yi Xue Ban.

[15] Zhang XY, Huang Z, Li QJ, Zhong GQ, Meng JJ, Wang DX (2020). Role of HSP90 in suppressing TLR4-mediated inflammation in ischemic postconditioning. Clin Hemorheol Microcirc.

[16] Wang DX, Huang Z, Li QJ, Zhong GQ, He Y, Huang WQ (2020). Involvement of HSP90 in ischemic postconditioning-induced cardioprotection by inhibition of the complement system, JNK and inflammation. Acta Cir Bras.

[17] Cheng XF, He ST, Zhong GQ, Meng JJ, Wang M, Bi Q (2023). Exosomal HSP90 induced by remote ischemic preconditioning alleviates myocardial ischemia/reperfusion injury by inhibiting complement activation and inflammation. BMC Cardiovasc Disord.

[18] He J, Huang L, Sun K, Li J, Han S, Gao X (2024). Oleuropein alleviates myocardial ischemia-reperfusion injury by suppressing oxidative stress and excessive autophagy via TLR4/MAPK signaling pathway. Chin Med.

[19] Yuan X, Juan Z, Zhang R, Sun X, Yan R, Yue F (2020). Clemastine fumarate protects against myocardial ischemia reperfusion injury by activating the TLR4/PI3K/Akt signaling pathway. Front Pharmacol.

[20] Shimamoto A, Chong AJ, Yada M, Shomura S, Takayama H, Fleisig AJ (2006). Inhibition of Toll-like receptor 4 with eritoran attenuates myocardial ischemia-reperfusion injury. Circulation.

[21] Chen X, Li X, Zhang W, He J, Xu B, Lei B (2018). Activation of AMPK inhibits inflammatory response during hypoxia and reoxygenation through modulating JNK-mediated NF-κB pathway. Metabolism.

[22] Zeng JJ, Shi HQ, Ren FF, Zhao XS, Chen QY, Wang DJ (2023). Notoginsenoside R1 protects against myocardial ischemia/reperfusion injury in mice via suppressing TAK1-JNK/p38 signaling. Acta Pharmacol Sin.

[23] Hu XX, Fu L, Li Y, Lin ZB, Liu X, Wang JF (2015). The cardioprotective effect of vitamin E (alpha-tocopherol) is strongly related to age and gender in mice. PLoS One.

[24] Gáspár R, Pipicz M, Hawchar F, Kovács D, Djirackor L, Görbe A (2016). The cytoprotective effect of biglycan core protein involves Toll-like receptor 4 signaling in cardiomyocytes. J Mol Cell Cardiol.

[25] National Research Council (US) Committee for the Update of the Guide for the Care and Use of Laboratory Animals (2011). Guide for the Care and Use of Laboratory Animals.

[26] Kiani AK, Pheby D, Henehan G, Brown R, Sieving P, Sykora P (2022). Ethical considerations regarding animal experimentation. J Prev Med Hyg.

[27] Mokhtari B, Azizi Y, Rostami Abookheili A, Aboutaleb N, Nazarinia D, Naderi N (2020). Human amniotic membrane mesenchymal stem cells-conditioned medium attenuates myocardial ischemia-reperfusion injury in rats by targeting oxidative stress. Iran J Basic Med Sci.

[28] Wang D, He S, Zhong G, Meng J, Bi Q, Tu R (2023). Effects of heat shock protein 90 on complement activation in myocardial ischemia/reperfusion injury after pioglitazone preconditioning. Adv Clin Exp Med.

[29] He ST, Wang DX, Meng JJ, Cheng XF, Bi Q, Zhong GQ (2022). HSP90-mediates liraglutide preconditioning-induced cardioprotection by inhibiting C5a and NF-κB. J Invest Surg.

[30] Gao M, Cai Q, Si H, Shi S, Wei H, Lv M (2022). Isoliquiritigenin attenuates pathological cardiac hypertrophy via regulating AMPKα in vivo and in vitro. J Mol Histol.

[31] Zhang XD, Sun GX, Guo JJ, Hu CC, Sun RC, Yu HC (2021). Effects of PPARγ agonist pioglitazone on cardiac fibrosis in diabetic mice by regulating PTEN/AKT/FAK pathway. Eur Rev Med Pharmacol Sci.

[32] Janssen W, Schwarz T, Bütehorn U, Steinke W, Sandmann S, Lang D (2022). Pharmacokinetics and mass balance of vericiguat in rats and dogs and distribution in rats. Xenobiotica.

[33] Najafi M (2013). Effects of postconditioning, preconditioning and perfusion of L-carnitine during whole period of ischemia/ reperfusion on cardiac hemodynamic functions and myocardial infarction size in isolated rat heart. Iran J Basic Med Sci.

[34] Ding Z, Liu X, Jiang H, Zhao J, Temme S, Bouvain P (2024). A refined TTC assay precisely detects cardiac injury and cellular viability in the infarcted mouse heart. Sci Rep.

[35] Resnick-Silverman L (2021). Using TUNEL assay to quantitate p53-induced apoptosis in mouse tissues. Methods Mol Biol.

[36] Harshitha R, Arunraj DR (2021). Real-time quantitative PCR: A tool for absolute and relative quantification. Biochem Mol Biol Educ.

[37] Cañada-García D, Arévalo JC (2023). A simple, reproducible procedure for chemiluminescent Western blot quantification. Bio Protoc.

[38] Heidenreich PA, Bozkurt B, Aguilar D, Allen LA, Byun JJ, Colvin MM (2022). 2022 AHA/ACC/HFSA guideline for the management of heart failure: Executive summary: A report of the american college of cardiology/american heart association joint committee on clinical practice guidelines. Circulation.

[39] McDonagh TA, Metra M, Adamo M, Gardner RS, Baumbach A, Böhm M (2022). 2021 ESC guidelines for the diagnosis and treatment of acute and chronic heart failure: Developed by the task force for the diagnosis and treatment of acute and chronic heart failure of the European society of cardiology (ESC) With the special contribution of the Heart Failure Association (HFA) of the ESC. Eur J Heart Fail.

[40] Tu RH, Wang DX, Zhong GQ, Meng JJ, Wen H (2021). New targets of morphine postconditioning protection of the myocardium in ischemia/reperfusion injury: Involvement of HSP90/Akt and C5a/NF-κB. Open Med (Wars).

[41] Wei H, Zhang Y, Jia Y, Chen X, Niu T, Chatterjee A (2024). Heat shock protein 90: Biological functions, diseases, and therapeutic targets. MedComm.

[42] Ranek MJ, Stachowski MJ, Kirk JA, Willis MS (2018). The role of heat shock proteins and co-chaperones in heart failure. Philos Trans R Soc Lond B Biol Sci.

[43] Kawai T, Ikegawa M, Ori D, Akira S (2024). Decoding toll-like receptors: Recent insights and perspectives in innate immunity. Immunity.

[44] Yu L, Feng Z (2018). The role of toll-like receptor signaling in the progression of heart failure. Mediators Inflamm.

[45] Yang Y, Lv J, Jiang S, Ma Z, Wang D, Hu W (2016). The emerging role of Toll-like receptor 4 in myocardial inflammation. Cell Death Dis.

[46] Edfeldt K, Swedenborg J, Hansson GK, Yan ZQ (2002). Expression of toll-like receptors in human atherosclerotic lesions: a possible pathway for plaque activation. Circulation.

[47] Nakamura K, Kageyama S, Kupiec-Weglinski JW (2019). Innate immunity in ischemia-reperfusion injury and graft rejection. Curr Opin Organ Transplant.

[48] Steenbergen C (2002). The role of p38 mitogen-activated protein kinase in myocardial ischemia/reperfusion injury; relationship to ischemic preconditioning. Basic Res Cardiol.

[49] Aamani N, Bagheri A, Masoumi Qajari N, Malekzadeh Shafaroudi M, Khonakdar-Tarsi A (2022). JNK and p38 gene and protein expression during liver ischemia-reperfusion in a rat model treated with silibinin. Iran J Basic Med Sci.

[50] Krishna M, Narang H (2008). The complexity of mitogen-activated protein kinases (MAPKs) made simple. Cell Mol Life Sci.

[51] Su Y, Zhao L, Lei D, Yang X (2024). Inhibition of circ_0073932 attenuates myocardial ischemia‒reperfusion injury via miR-493-3p/FAF1/JNK. In Vitro Cell Dev Biol Anim.

[52] Xu T, Zhang K, Kan F, Li F, Yu B, Du W (2019). Adeno-associated virus 9-mediated small RNA interference of TLR4 alleviates myocardial ischemia and reperfusion injury by inhibition of the NF-κB and MAPK signaling pathways in rats. Curr Mol Med.

[53] Chang C, Hu L, Sun S, Song Y, Liu S, Wang J (2021). Regulatory role of the TLR4/JNK signaling pathway in sepsisinduced myocardial dysfunction. Mol Med Rep.

[54] Xu H, Yao Y, Su Z, Yang Y, Kao R, Martin CM (2011). Endogenous HMGB1 contributes to ischemia-reperfusion-induced myocardial apoptosis by potentiating the effect of TNF-&alpha;/JNK. Am J Physiol Heart Circ Physiol.

[55] Yang Z, Xie Y, Li M, Chen W, Zhong C, Ju J (2024). Ramelteon alleviates myocardial ischemia/reperfusion injury (MIRI) through Sirt3--dependent regulation of cardiomyocyte apoptosis. Biomed Pharmacother.

[56] Hu B, Tian T, Li XT, Hao PP, Liu WC, Chen YG (2023). Dexmedetomidine postconditioning attenuates myocardial ischemia/reperfusion injury by activating the Nrf2/Sirt3/SOD2 signaling pathway in the rats. Redox Rep.

[57] Yovas A, Manjusha WA, Ponnian SMP (2022). β-caryophyllene modulates B-cell lymphoma gene-2 family genes and inhibits the intrinsic pathway of apoptosis in isoproterenol-induced myocardial infarcted rats; A molecular mechanism. Eur J Pharmacol.

[58] Korshunova AY, Blagonravov ML, Neborak EV, Syatkin SP, Sklifasovskaya AP, Semyatov SM (2021). BCL2regulated apoptotic process in myocardial ischemiareperfusion injury (Review). Int J Mol Med.

[59] Hasan A, Haque E, Hameed R, Maier PN, Irfan S, Kamil M (2020). Hsp90 inhibitor gedunin causes apoptosis in A549 lung cancer cells by disrupting Hsp90: Beclin-1: Bcl-2 interaction and down-regulating autophagy. Life Sci.

